# Rapid LC-MS/MS Evaluation of Collagen and Elastin Crosslinks in Human and Mouse Lung Tissue with a Novel Bioanalytical Surrogate Matrix Approach

**DOI:** 10.3390/ijms252313026

**Published:** 2024-12-04

**Authors:** Sarah M. Lloyd, Elizabeth J. Sande, Kenneth Ruterbories, Stephen P. O’Brien, Yue-Ting Wang, Lucy A. Phillips, Tracy L. Carr, Meghan Clements, Lisa A. Hazelwood, Yu Tian, Yupeng He, Qin C. Ji

**Affiliations:** 1 AbbVie Inc., 1 North Waukegan Rd., North Chicago, IL 60064, USA; 2 AbbVie Bioresearch Center, 100 Research Drive, Worcester, MA 01605, USA

**Keywords:** crosslinking, collagen, elastin, lung, fibrosis, LC-MS/MS, surrogate matrix, bioanalysis

## Abstract

Alterations to post-translational crosslinking modifications in the extracellular matrix (ECM) are known to drive the pathogenesis of fibrotic diseases, including idiopathic pulmonary fibrosis (IPF). Thus, the methodology for measuring crosslinking dynamics is valuable for understanding disease progression. The existing crosslinking analysis sample preparation and liquid chromatography tandem mass spectrometry (LC-MS/MS) methods are typically labor-intensive and time-consuming which limits throughput. We, therefore, developed a rapid approach minimizing specialized equipment and hands-on time. The LC-MS/MS sample analysis time was reduced to two minutes per sample. We then improved the analytical integrity of the method by developing a novel surrogate matrix approach for the dihydroxylysinonorleucine (DHLNL) crosslink. By modifying sample preparation, we prepared a tissue-based surrogate matrix with undetectable levels of endogenous DHLNL, providing a strategy for quantifying this crosslink with a more relevant standard matrix. We then applied this rapid methodology to evaluating crosslinking in lung fibrosis. We showed an increase in DHLNL in human IPF lung relative to healthy donors, as well as in a fibrotic mouse model. Finally, we demonstrated that this increase in DHLNL could be mitigated with an anti-fibrotic compound, suggesting that this assay has potential for evaluating pharmaceutical compound efficacy.

## 1. Introduction

**Figure 1:**
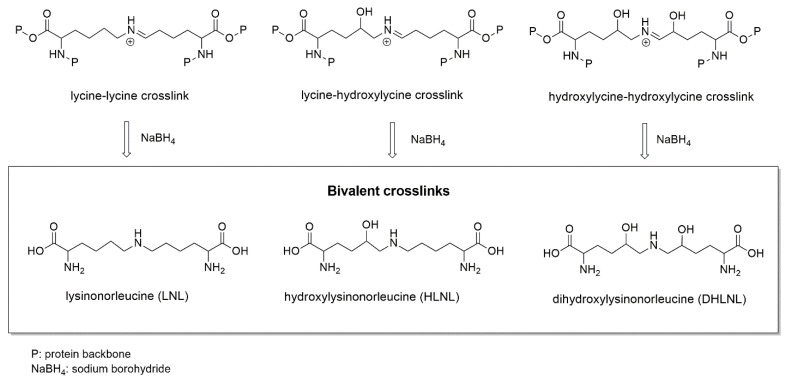
Structures of bivalent crosslinks analyzed by LC-MS/MS, and their precursors (intermediate imine crosslink structures). Bivalent crosslinks can only be analyzed after their unstable precursors are reduced with sodium borohydride (NaBH_4_).

**Figure 2:**
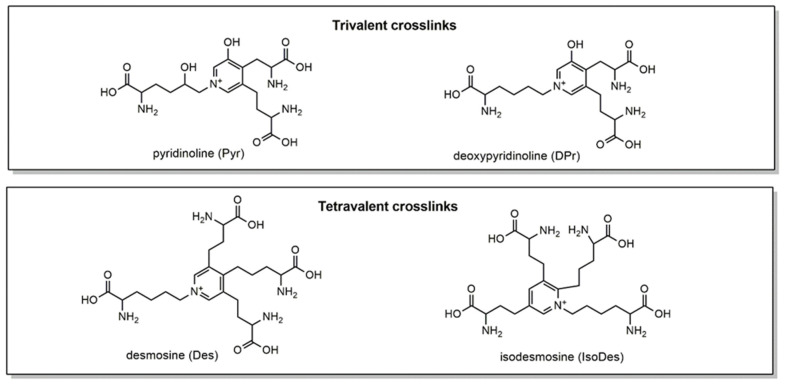
Structures of trivalent and tetravalent crosslinks analyzed by LC-MS/MS. Pyr and DPr crosslinks are found on collagen, while Des and IsoDes are specific to elastin.

Extracellular matrix (ECM) proteins create an intricate structural meshwork in between cells important for physical support and cellular signaling [1]. Crosslinking modifications to these ECM proteins alter their structure, function, and susceptibility to enzymatic degradation [2]. Changes to overall crosslinking composition and abundance have been observed in fibrotic disease [3,4,5] and cancer [6], as well as in studies of reproductive biology [7,8] and aging [9]. The crosslinking dynamics in collagen and elastin proteins are of high interest. Collagen contains both bivalent immature and trivalent mature crosslinks [2]. Immature bivalent crosslinking modifications of collagen include lysinonorleucine (LNL), hydroxylysinonorleucine (HLNL), and dihydroxylysinonorleucine (DHLNL) (Figure 1). Notably for crosslinking analysis by LC-MS/MS, these immature collagen crosslinks must be stabilized by a reduction reaction with sodium borohydride (NaBH_4_) (Figure 1) [9,10]. Immature crosslinks can go through additional reactions to form mature trivalent collagen crosslinks: pyridinoline (Pyr) and deoxypyridinoline (DPr) (Figure 2) [2]. On elastin, tetravalent crosslinks, desmosine (Des) and isodesmosine (IsoDes), are formed (Figure 2).

Liquid chromatography tandem mass spectrometry (LC-MS/MS) serves as a sensitive, selective, and quantitative method for evaluating crosslinking. A number of groups have successfully implemented methodology for the simultaneous detection of collagen and elastin crosslinks [6,7,8,9,10,11,12]. These methods typically require advanced equipment and special expertise for sample preparation. Furthermore, protocols for sample preparation, as well as LC-MS/MS analysis, can be time-consuming. For example, the lyophilization of tissue is included in a number of published methods which can take up to a day and requires lyophilization equipment [3,8,10,12,13,14]. Solid-phase extraction (SPE) is also a standard method included in sample preparation but can similarly be time-consuming, and requires both technical skills and equipment [7,10,12,13,15]. Beyond sample preparation steps, acquisition time per sample on LC-MS/MS is a limiting factor for sample throughput. Naffa et al. succeeded in reducing the total run time to approximately ten minutes per sample [10]. Similarly, other methods measuring three-to-four crosslink analytes still have sample run times greater than 5 min [8,16]. While these methods are improvements in crosslink analysis, limitations in efficiency and throughput remain.

Gaps in the quantification of LC-MS/MS crosslinking analysis also remain. For the best quantitative readout, an appropriate matrix for calibration standards must be chosen. The matrix used influences the performance and sensitivity of analytical assays. Biological matrices are confounded by endogenous levels of analytes of interests and components that may interfere with results [17]. It is, therefore, optimal to match the matrix for analyte standards with that of the samples being tested. For endogenous analytes such as ECM crosslinks, however, this can be difficult since endogenous levels of an analyte alter detection and sensitivity [18]. For existing crosslink analysis methodologies, calibration curves have been generated with non-biological matrices rather than a tissue-based matrix [9,10]. Although this does not represent tissue samples prepared for analysis, it can be sufficient for the purpose of relative quantitative comparisons; however, advances in matrix selection could allow for a more reliable, quantitative measurement of crosslinking with LC-MS/MS.

Due to the limitations of existing methods, we sought to develop an improved methodology for the LC-MS/MS analysis of crosslinking modifications with a novel tissue matrix calibration curve. We simplified sample preparation significantly, reducing the specialized equipment and hands-on time required for sample preparation. We further optimized the LC-MS/MS analysis by reducing the acquisition time per sample to only two minutes. To assess instrument and method performance, we incorporated calibration standards and quality control samples for analytes using water as a surrogate matrix. In addition, for the potential future need of quantitative analysis, we developed a novel quantification method for DHLNL using a more relevant surrogate matrix derived from human tissue. To our knowledge, no quantitative assay performance evaluation of this kind has been reported in crosslinking peptide product analysis. We then applied this advanced methodology and showed changes in crosslinking in fibrotic lungs consistent with recently published results. We further demonstrated that crosslinking methodology can be used as an effective readout for anti-fibrotic compound efficacy. Overall, we developed a robust, simplified, and highly efficient methodology for LC-MS/MS crosslinking analysis and demonstrated that this powerful tool can be used for pharmaceutical applications.

## 2. Results

### 2.1. Tissue Sample Preparation

Crosslink analysis by LC-MS/MS involves complex sample preparation protocols, followed by lengthy LC-MS/MS runs [10]. To develop an optimized, rapid methodology for crosslinking analysis, we used the human lung as a model system. Crosslinking has been shown in recent LC-MS/MS-based studies to be dysregulated in idiopathic pulmonary fibrosis (IPF) [3,4]. Thus, we paired methodological advancements in crosslink analysis with relevant biological application. For sample preparation and LC-MS/MS method development, we used lungs from three healthy and three IPF donors and took three fragments from each lung for crosslinking analysis. The lungs were also assessed via histology (Supplementary Figure S1).

A typical crosslink analysis of tissue samples begins with 5–10 mg [7,13,15] of tissue. Often, samples are freeze-dried [3,8,10,12,13]; however, tissue lyophilization protocols can take nearly twenty-four hours and require access to freeze-drying equipment [14]. Alternatively, tissue samples can be homogenized without the freeze-drying step [15]. This provides flexibility in samples that can be utilized and allows for rapid tissue processing. Thus, for this method, we applied tissue homogenization rather than lyophilization. Flash-frozen tissues were homogenized in only three minutes. This was sufficient to break down even fibrous lung tissue such as that from donors with IPF.

**Figure 3:**
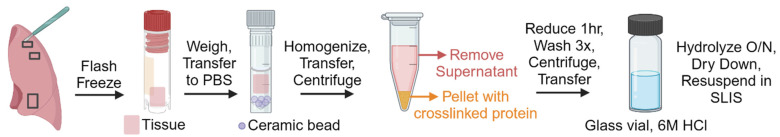
Overview of the crosslinking sample preparation procedure. Three fragments per lung for three healthy and three IPF donors were used for method development. Tissue was homogenized and centrifuged. The pellet contained crosslinked ECM. NaBH_4_ reduction was performed, followed by three washes. Samples were hydrolyzed overnight (O/N) in HCl, dried down, and resuspended in a stable labeled internal standard (SLIS). Created with BioRender.com, accessed 8 July 2024.

After homogenization, samples were centrifuged and crosslinked protein was collected in the pellet. At this step, supernatant was removed. The sample was then reduced, washed three times, and hydrolyzed. Typically, prior to LC-MS/MS analysis, hydrolyzed samples are enriched by solid-phase extraction [7,10,12,13,15]. This is carried out with a cellulose column which can be purchased ready for use or hand-packed by the user. Including this step adds significant time to sample preparation. To improve efficiency and accessibility, we proceeded without the solid-phase extraction step. Quality control checks of this method by LC-MS/MS in subsequent sections will demonstrate that crosslinks were still clearly detectable and appropriately separated. Furthermore, our biological application of the method provided consistent results with the recent literature reports. Thus, we developed a streamlined sample preparation method using limited specialized equipment that can be completed with no solid-phase extraction (Figure 3).

### 2.2. Chromatography and Mass Spectrometry

Crosslink analytes are notoriously difficult to analyze due to their small, polar, charged, and structurally similar components (Figure 1 and Figure 2). In the literature, as many as twenty crosslink analytes have been measured simultaneously using the hydrophilic interaction liquid chromatography (HILIC) approach; however, this comes with the price of at least twenty minutes per sample [9]. For balance, we sought to develop an optimized, faster method for the simultaneous analysis of key crosslinks using reverse-phase chromatography. This came with challenges including retention, column availability, peak resolution, and chromatographic peak quality.

First, an ion-pairing mobile phase reagent, heptafluorobutyric acid (HFBA), in water was tested for gaining polar retention on the column. This aqueous mobile phase has been used in published literature for crosslink analysis and is a popular choice for very polar compounds [16,19]. This mobile phase is on a dedicated AB Sciex 5500 instrument (Framingham, MA, USA) in the laboratory utilizing atmospheric pressure chemical ionization (APCI). While retention was achieved with Waters XBridge C18, the signal for even the neat D4-DHLNL stable labeled internal standard (SLIS) was low, and we aimed to improve the signal intensity (Supplementary Figure S2).

As an alternative, we tested electrospray ionization (ESI) with an identical mobile phase on an AB Sciex 6500+ instrument to gain the best possible sensitivity. Ion source temperatures were optimized to ensure maximum signal for the crosslink analytes. We tested a range from 600 to 725 °C, with 725 °C having the highest intensity for all crosslinks. The biggest challenge for this method was the peak resolution of the closely related crosslink structures, especially for co-eluting Pyr and DPr. The Waters XBridge C18 5 µm 2.1 × 30 mm was able to retain and elute all crosslinks within one minute, but the resolution between Pyr and DPr was too poor at R = 0.33, meaning that they almost co-eluted. Even with flattening the slope of the gradient, these peaks could not be baseline-resolved with this column choice without compromising the peak shape of the earliest eluting peak, DHLNL.

**Figure 4:**
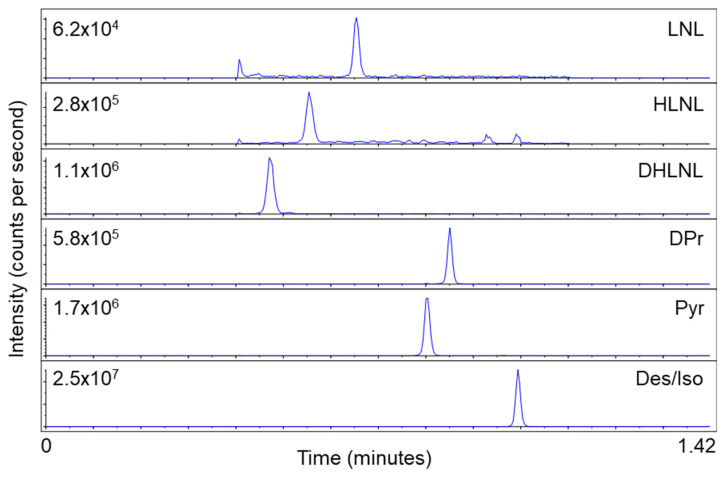
Chromatographic elution order, peak resolution, and signal intensities of six crosslinks from a lung tissue sample with the two minute per sample method. Des/Iso represents desmosine and isodesmosine, as these were not separable.

To increase the integrity of the ESI method, we screened additional columns to establish a baseline resolution (R) of around R = 1.0 for all crosslinks. Additional column screening included the longer and 100% aqueous stable Waters Atlantis T3 5 µm 2.1 × 50 mm column; however, the resolution was R < 0.30 for Pyr and DPr. The second 100% aqueous stable column used was the Phenomenex EVO C18 2.6 µm 2.1 × 50 mm. This column, with a flattened gradient, offered the resolution of R = 0.80 for Pyr and DPr, which was a vast improvement over previous columns and LC conditions. The method was optimized for one minute total run time, but the desmosine and isodesmosine crosslink pair (Des/Iso) eluted after the gradient time ended (retention time > 0.80 min). To avoid eluting any analytes of interest in this end-portion of the method, the gradient time was extended from 0.60 min to 0.80 min. This change allowed for all crosslinks to elute over 0.80 min using 10–28% organic mobile phase B, acetonitrile. Finally, the Waters Cortecs C18 2.6 µm 2.1 × 50 mm column was chosen for its very similar retention and resolution with the same LC parameters, but superior sharp peak shape. An analyst was used for peak integration. The optimized elution gradient conditions are shown in Table 1. Multiple reaction monitoring (MRM) transitions, collision energy, and declustering potential were optimized individually and are shown in Table 2. Altogether, this resulted in each sample requiring only two minutes for the robust, simultaneous detection of LNL, HLNL, DHLNL, Pyr, DPr, and Des/Iso (Figure 4).

### 2.3. LC-MS/MS Quantification

For assay analysis, we first generated calibration curves for each analyte using water as a surrogate matrix. Calibration curves were prepared for all analytes of interest except for HLNL due to the unavailability of a standard. The standard lower limit of quantitation (LLOQ) was approximately 1 ng/mL for all analytes, except LNL which was 5 ng/mL. Although water served as an effective matrix for calibration curves to report the relative peak area ratio of the peak signal to the internal standard peak area for all analytes, we sought to establish a more relevant matrix. Ideally, standards would be added to a blank sample matrix at the beginning of sample preparation to assess recovery and allow for quantification in the true sample matrix. Because the sample preparation uses the insoluble fraction, however, these solubilized standards could not be spiked in at beginning of sample preparation. Additionally, crosslinked proteins are fundamental and present across all body tissues. Thus, there would be a high baseline of endogenous crosslinks in any tissue sample measured. To overcome these challenges, we developed a novel strategy to quantify a critical crosslink, DHLNL, using a surrogate matrix generated from human tissue.

**Figure 5:**
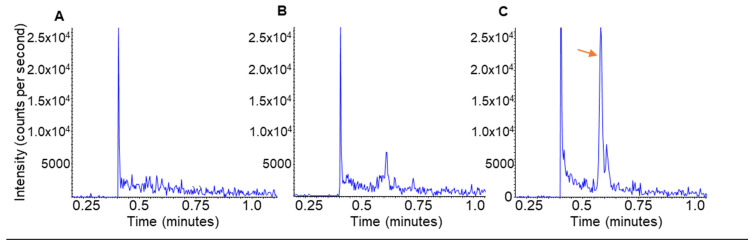
Development of a relevant surrogate matrix for DHLNL quantitation. Chromatograms of (**A**) the neat water blank used for the standard generated calibration curves; (**B**) non-reduced lung tissue, blank DHLNL matrix; and (**C**) 2.5 ng/mL DHLNL (LLOQ) in an unreduced tissue-based surrogate matrix. The orange arrow points to the DHLNL peak at a retention time of 0.58 min.

To measure DHLNL from tissue, it must be reduced by NaBH_4_ during sample preparation. We posited that removing the reduction step from sample preparation would generate tissue matrix with minimally measurable DHLNL. When lung tissue was prepared without a NaBH_4_ reduction, endogenous DHLNL in reduced form was not present, providing a clean lung homogenate matrix for establishing proper analytical criteria to report absolute concentrations in tissue samples. The surrogate matrix calibration curve standards were diluted 10×, as was performed with the study samples. After the correct dilution factors were applied to each DHLNL calibration curve (Table 3), the neat and surrogate matrix curves showed equivalency within ±20% accuracy of the target concentrations and ≤15% precision between respective replicates. Calibration curves generated with this method revealed an LLOQ of 2.5 ng/mL, which gives a 5:1 signal-to-noise ratio when this standard is compared to the matrix blank (Figure 5). This novel preparation provides an option for the quantitative evaluation of DHLNL using a more relevant, tissue-based matrix. This advanced tissue-based matrix approach highlights the analytical integrity of this method, giving confidence in its potential for biological applications. Future work should include researching ways to apply this surrogate matrix technique to all other crosslinks to report the absolute quantities in tissue samples, as it was only successful for DHLNL.

### 2.4. Evaluation of Normalization Strategies

Next, we evaluated crosslinking from a biological perspective. To demonstrate the differences between diseased and healthy lungs, we applied relative quantification. The peak area of each analyte was calculated and evaluated across conditions. Due to variations in starting tissue size, however, data normalization was required to assess biological differences across samples. The common practice is to normalize crosslink levels by total hydroxyproline, which can be measured by chromatography or a colorimetric assay [3,4,20]. Chromatography-based hydroxyproline measurement adds an additional analyte to LC-MS/MS analysis and requires the acquisition of a hydroxyproline standard [3]. While hydroxyproline colorimetric assays are relatively simple to run, they take several hours and can be costly, at greater than five-hundred US dollars per plate. Alternatively, total protein in sample can be measured by a standard colorimetric assay and used for normalization [20]. In the interest of time and cost efficacy, we asked how hydroxyproline normalization compared to other strategies.

**Figure 6:**
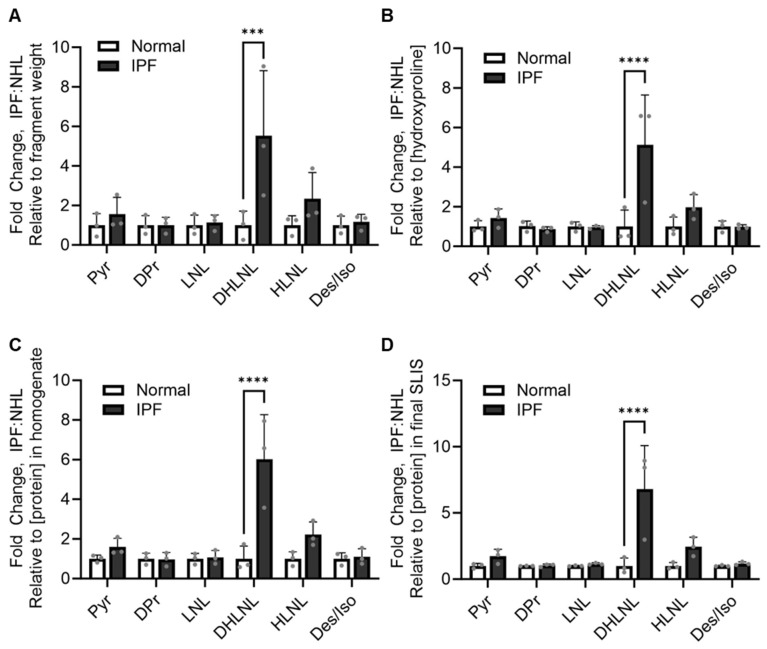
Trends in crosslinking changes are consistent across normalization methods. Values from the LC-MS/MS relative quantification of pyridinoline (Pyr), deoxypyridinoline (Dpr), lysinonorleucine (LNL), hydroxylysino-norleucine (HLNL), dihydroxylysino-norleucine (DHLNL), and desmosine/isodesmosine (Des/Iso) were normalized by (**A**) tissue fragment weight, (**B**) hydroxyproline concentration, (**C**) total protein concentration of homogenized tissue and (**D**) total protein concentration of the final sample in the stable label internal standard (SLIS). Relative values were then used to calculate the fold change of samples relative to normal healthy control donors. Data are shown as the mean with standard deviation. Each dot represents a biological replicate *(n* = 3 IPF donor lungs, *n* = 3 normal healthy lungs (NHL)) and is an average of three technical replicates (*n* = 3 fragments/donor lung). Statistics are shown for all comparisons, where *p* < 0.05 (two-way ANOVA, Sidak’s multiple-comparison test, **** *p* < 0.0001, *** *p* < 0.001).

We assessed the crosslinking differences in IPF relative to normal healthy lungs with four different normalization methods: fragment weight, hydroxyproline concentration of hydrolysate, total protein concentration of homogenized tissue, and total protein concentration of the final sample in SLIS. The relative quantification values from LC-MS/MS were normalized by each of these methods. Then, fold change was calculated relative to normal healthy donors. All normalization methods showed comparable trends in crosslinking changes. The immature crosslink DHLNL was increased in IPF across all normalization methods (Figure 6). Importantly, this is consistent with data published by Jones et al., showing a strong increase in DHLNL in IPF when normalized with hydroxyproline [4]. This suggests that the total protein concentration could serve as a fast, low-cost alternative to hydroxyproline measurement. Additionally, the consistency between these data and published works provides strong confidence in the reliability of this method.

### 2.5. Application to Pharmaceutical Research

To provide additional confidence in the developed method and demonstrate utility for biological research, we investigated crosslinking in the bleomycin mouse model of lung fibrosis. Recently, using standard crosslinking LC-MS/MS methodology, Ma et al. showed significant increases in DHLNL in a bleomycin mouse model [3]. We aimed to determine if the same results could be reproduced with our advanced methodology. We also hypothesized that crosslinking could serve as a readout for drug efficacy, which could be promising for evaluating therapeutics. To test this, we injected mice with bleomycin and seven days later, treated them with a vehicle control or SM16, a known antifibrotic compound which inhibits the TGF-beta pathway (*n* = 5 mice/condition) [21]. The lungs were collected and processed for crosslinking analysis with our developed method.

**Figure 7:**
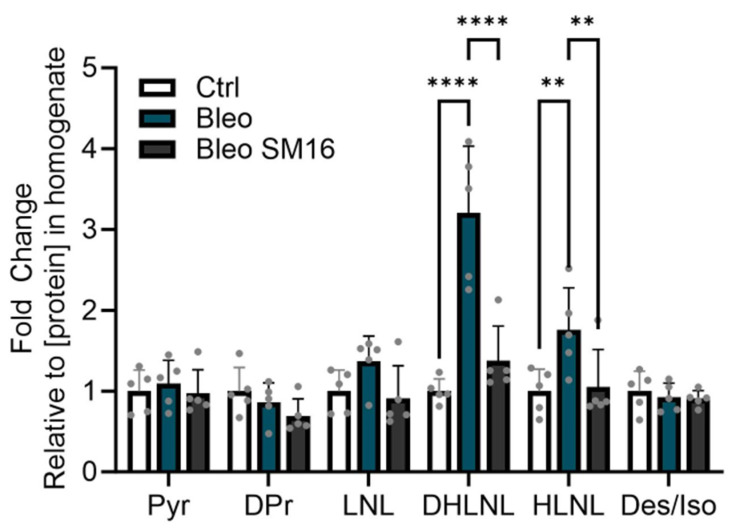
DHLNL crosslinking increases in bleomycin mouse model of lung fibrosis and decreases with antifibrotic compound. Values from the LC-MS/MS relative quantification of pyridinoline (Pyr), deoxypyridinoline (Dpr), lysinonorleucine (LNL), hydroxylysinonorleucine (HLNL), dihydroxylysinonorleucine (DHLNL), and desmosine/isodesmosine (Des/Iso) were normalized by total protein concentration in the homogenate. Relative values were then used to calculate the fold change of the samples relative to the control (Ctrl) which had no bleomycin (Bleo) or SM16 treatment. The Bleo condition had bleomycin but no SM16 treatment. The SM16 group had both bleomycin and SM16 treatment. Data are shown as the mean with standard deviation. Each dot represents a biological replicate (*n* = 5 mice/condition). Statistics are shown for all comparisons where *p* < 0.05 (two-way ANOVA, Tukey’s multiple comparison test, **** *p* < 0.0001, ** *p* < 0.01).

Consistent with published work [3], bleomycin induced an increase in DHLNL (Figure 7). This is also consistent with the increase in DHLNL seen in human tissue, both in this study and in the literature [4]. With SM16, this increase in DHLNL was strongly reduced (Figure 7). Interestingly, this model also showed an increase in HLNL with bleomycin which was decreased with SM16. These data suggest that this highly efficient methodology successfully captures biological changes in crosslinking. Furthermore, this more accessible method can be applied to biological applications such as fibrosis research, where crosslinking can be a central driver of disease. This additionally provides an interesting and relevant endpoint for evaluating compound efficacy in preclinical models for pharmaceutical applications.

## 3. Discussion

We developed an improved, highly efficient methodology for evaluating collagen crosslinking in the context of human disease. We reduced both the time and specialized equipment required for LC-MS/MS crosslinking studies. With this method, any group could feasibly flash-freeze tissues of interest for crosslinking studies without the requirement of lyophilization equipment. This strategy could be modified to work with additional tissue types or different bead-based homogenizers, making it highly accessible to most labs that process tissue samples regularly. This is valuable given that crosslinking dynamics are relevant to a wide variety of diseases in addition to fibrosis, including cancer, aging, and reproductive health [6,7,8,9]. By also removing SPE columns in sample preparation, almost all sample preparation can be carried out with standard laboratory equipment. Thus, it is feasible for almost all of sample preparation to be completed by biologists collecting tissue samples rather than requiring those with specialized expertise and equipment. This provides flexibility for collaborative investigations spanning biology with bioanalytical studies.

For biological studies of human disease, sample availability can often be a limiting factor. This developed methodology allows for maximizing the usage of tissue samples. It only requires 5–10 mg of tissue for successful crosslinking measurement in human and mouse lungs. Thus, one lung could be used to generate many flash-frozen fragments to be used for matched experiments. Transcriptomic, proteomic, and crosslinking readouts from a single lung would be possible. This methodology also allows for multiple assays to be run from a single chunk of tissue. The supernatant from homogenized tissue could be used for other biological assays evaluating soluble proteins such as ELISA.

In addition to improving efficiency, we demonstrated the quantitative potential of this LC-MS/MS method. The quantification strategy can be chosen based on study requirements. For example, for comparison across healthy and diseased lung, the relative area ratio quantification was sufficient. This effectively demonstrated the differences between treatment groups. For bioanalytically focused approaches, however, absolute quantification can be performed with appropriate standards. This is dependent on the availability of and access to pure standards, which can pose a limitation [22]. For method evaluation, we generated calibration curves with a non-tissue-based surrogate matrix similar to previous reports which have used 0.1% formic acid or water [10]. This was carried out for all standards except HLNL due to the limitation of standard availability. Thus, this rapid LC-MS/MS method is amenable to absolute quantitation, but simple peak area ratio calculations may also be sufficient for comparing between groups.

Furthermore, we demonstrated that a number of simple normalization strategies can be applied for quantification. We achieved normalization by the total collagen content based on a hydroxyproline colorimetric assay which is common practice in crosslinking normalization. While this method is simple, it can be expensive. Additionally, total collagen increases in fibrosis, so we explored other options. One alternative was normalization by fragment weight. This strategy can create more variability since precision is influenced by the moisture in tissue. This does not account for the altered density of diseased tissue. To improve precision, we normalized by the total protein content which is a cost- and time-efficient method. We measured total protein in the supernatant after homogenization, as well as in the final sample in SLIS. While both of these methods provided similar results, using values from the final sample in SLIS may be the most accurate since it is the final form of the sample run on LC-MS/MS. This accounts for any material lost during sample preparation. Finally, an option which was not addressed here is also monitoring hydroxyproline in the LC-MS/MS assay.

Another important consideration for quantification is selecting an appropriate matrix for calibration curve generation. Existing crosslinking analysis methodologies have applied non-biological matrices, different from that which samples are in for calibration curve generation [10]. Ideally, for calibration, however, it is best for the matrix to be consistent with that of the samples. The challenge of this is the endogenous levels of analytes interfering with quantification [17,18]. To generate a more relevant matrix for quantification, we devised a novel method using a truer surrogate matrix. We leveraged sample preparation chemistry and removed the NaBH_4_ reduction step to create a tissue-based matrix absent of DHLNL. We made calibration curves with this matrix and showed that DHLNL could be quantified in an unreduced, tissue-based matrix. Thus, our methodology is amenable to standard quantification with a non-biological surrogate matrix, and DHLNL, a crucial crosslink in fibrosis, can be quantified in a tissue-based matrix. Overall, we provided an improved, rapid LC-MS/MS strategy for evaluating collagen crosslinking. This method increases accessibility by reducing the need for specialized training and equipment. Both hands-on sample preparation time and total time on LC-MS/MS were decreased. We showed that this method allows for quantification and developed a novel surrogate matrix strategy for DHLNL. Lastly, we demonstrated the utility of this methodology for biological research. We highlighted critical changes in crosslinking in lung fibrosis and showed the potential for this method to serve as a readout for therapeutic compound efficacy.

## 4. Materials and Methods

### 4.1. Human Tissue Collection

Studies in this work abide by the Declaration of Helsinki principles. Human lungs were received from the International Institute for the Advancement of Medicine, an unincorporated division of the Musculoskeletal Transplant Foundation (IIAM) or from the National Disease Research Interchange (NDRI). Small sections of lung were flash-frozen in liquid nitrogen and stored at −80 °C until use.

### 4.2. Bleomycin Mouse Model

Twenty-eight 8-week-old male mice from Taconic (C57Bl/6N) were split into three groups randomized by body weight. Bleomycin and phosphate-buffered saline (PBS) was administered via oropharyngeal administration (OA) under light anesthesia, approximately 1.5 min, and dosed appropriately with a maximum volume of 50 µL. Eight mice were utilized as the negative controls aspirated with 50 μL of PBS. The remaining two groups, *n* = 10, each received 2.0 U/kg bleomycin. Alk5 inhibitor (SM16) A-1944199 was administered daily at 45 mg/kg for 14 days, from day 8 to 21 per os (PO). Diet gel nutrient supplement was supplied and the body weight was monitored throughout the study. At 21 days post-OA delivery, the animals were sacrificed, and lungs harvested and flash-frozen. Tissues from five animals from each condition were used for crosslinking analysis. Studies were approved by AbbVie IACUC.

### 4.3. Tissue Sample Preparation

Human or mouse lung tissue was weighed and placed in a tissue homogenizing tube with 1.4 mm ceramic beads (Omni International, Kennesaw, GA, USA, SKU:19-627) and 500 µL Dulbecco’s phosphate-buffered saline (dPBS). Tissue was homogenized using Bead Ruptor Elite Bead Mill Homogenizer (Omni International) rat lung protocol, 30 s on, 20 s off, 4 m/s three times. Homogenate was transferred to microcentrifuge tubes. Beads were washed with dPBS to collect any remaining homogenate. The homogenate and dPBS wash were combined and centrifuged at 18,000 rcf for 20 min at 4 °C. Supernatants were saved for total protein measurement with a detergent-compatible (DC) protein assay. Pellets were resuspended in 1 mg/mL NaBH_4_ in 0.1 N NaOH and placed on a rocker for 1 h at 4 °C. Glacial acetic acid was added to 0.1% and samples were centrifuged at 18,000 rcf for 20 min at 4 °C three times. Pellets were resuspended in 2 mL 6N HCl, transferred to glass vials (Chem Glass, Vineland, NJ, USA, CG-4912-01), and placed on a heat block at 110 °C overnight (16 h). HCl was removed by nitrogen down on a heat block set to 80 °C. Dried samples were resuspended in 500 µL of an internal standard solution for LC-MS/MS analysis. Th total protein of protein samples was measured using the DC Protein Assay Kit (Biorad, Hercules, CA, USA, 5000111). The hydroxyproline assay was measured with a QuickZyme Biosciences kit (QZBtishyp2, Leiden, The Netherlands).

### 4.4. Standard Preparation

Crosslink standards for LNL (Toronto Research Chemicals, Toronto, ON, Canada, TRC-L488750), DHLNL (Toronto Research Chemicals TRC-D452900), Pyr (BOC Sciences Shirley, NY, USA 63800-01-1), DPr (BOC Sciences, B2694-136861), and Des/IsoDes (EPC Inc., Owensville, MO, USA, DR44) were solvated to 1 mg/mL in a water/DMSO mixture (1:1 *v*/*v*), respectively. A mixture of 7 crosslinks at 10 µg/mL was prepared in water, then diluted down to the calibrators using water containing a stable isotope-labeled internal standards. D4-desmosine was used for Des/IsoDes, and D4-dihydroxylysinonorleucine was used for collagen crosslinks. The final concentrations of the calibrators were 1000, 500, 200, 100, 50, 20, 10, 5, 2, and 1 ng/mL.

### 4.5. Chromatographic Separation and MS/MS Detection

The mobile phases used in this reversed-phase method were A = 50 mM heptafluorobutyric (HFBA) acid in water and B = 100% acetonitrile. Chromatographic separation was achieved using a Waters Cortecs C18 column (2.7 µm, 2.1 × 50 mm) (Waters Corporation, Milford, MA, USA) using a 1.5 mL/min flow rate. The total run time to elute all crosslinks and SLIS was two minutes. The gradient elution ramps from 8 to 28% B over a total of 0.80 min (Table 1). The flow is diverted to the mass spectrometer from 0.4 to 1.1 min and to waste at all other times to maintain instrument performance. Analysis was performed on a SCIEX 6500+ triple–quadrupole mass spectrometer (AB Sciex LLC, Framingham, MA, USA) coupled with an Agilent 1290 UPLC system (Agilent Technologies, Inc., Santa Clara, CA, USA) and PAL autosampler (CTC Analytics AG, Zwingen, Switzerland). Analytes were detected using electrospray ionization (ESI) with multiple-reaction monitoring (MRM) in a positive mode.

### 4.6. Crosslink Sample Analysis

To evaluate the methodology, calibration curves were constructed for all analytes, except HLNL, and prepared in water as a neat matrix for system suitability and instrument evaluation during the analysis time. The peak area ratio versus the corresponding concentrations of the calibrators was plotted. Curves were fitted using a linear or quadratic fit with 1/x or 1/x^2^ weighting. For the evaluation of biological changes, the relative peak area ratio of the analyte versus internal standard response was used. The peak area ratios of each analyte were calculated using Analyst^®^ software (SCIEX) version 1.7. Values were normalized and compared between conditions (healthy vs. disease, treatment vs. control).

### 4.7. DHLNL Blank Surrogate Matrix Preparation

Sample preparation was completed with a healthy lung fragment, as described in ‘Tissue sample preparation’, except with removal of the NaBH_4_ reduction. The sample was incubated for 1 h in 0.1 N NaOH without NaBH_4_. This unreduced sample served as a blank surrogate matrix for DHLNL for use in absolute quantification. Calibrators were prepared by a matrix matching sample amount (10 µL surrogate matrix + 10 µL of working stock solution), then supplied with 80 µL of SLIS to bring the standard to volume. The LLOQ of this method was 2.5 ng/mL.

### 4.8. Software

GraphPad Prism 10.1.2., Analyst^®^ software (SCIEX) version 1.7 with HotFix 3 was used. Figure 3 was made with BioRender.com and exported under AbbVie’s paid subscription.

## Figures and Tables

**Table 1:** Elution gradient used for crosslink analyte separation.

Time (min)	A% (50 mM HFBA)	B% (Acetonitrile)	Flow (µL/min)
0	92	8	1500
0.2	92	8	1500
1	72	28	1500
1.01	2	98	1500
1.4	2	98	1500
1.42	92	8	1500

**Table 2:** Multiple reaction monitoring (MRM) conditions for the detection of crosslinks.

Q1 *m*/*z*	Q3 *m*/*z*	Analyte	Collision Energy
429.3	267.3	Pyridinoline	40
413.3	267.2	Deoxypyridinoline	35
526.4	397.3	Desmosine/Isodesmosine	40
276.3	85	Lysinonorleucine	35
308.3	128.1	Dihydroxylysinonorleucine	30
292.3	84	Hydroxylysinonorleucine	35
530.4	485.3	D4-Desmosine	45
312.3	130.1	D4-Dihydroxylysinonorleucine	30

**Table 3:** Neat and surrogate matrix standard equivalency for non-reduced DHLNL.

Standard	Concentration of the Neat Calibrator Solution (ng/mL)	Absolute Amount of Neat Standards (ng/0.1 mL) Using 96% SLIS	Absolute Amount of Surrogate Matrix Standards (ng/0.1 mL) Using 80% SLIS
S1	1000	96	7.68
S2	500	48	3.84
S3	200	19.2	1.536
S4	100	9.6	0.768
S5	50	4.8	0.384
S6	20	1.92	0.154
S7	10	0.96	0.077
S8	5	0.48	0.038
S9	2	0.192	0.015
S10	1	0.096	0.008

## Data Availability

The original contributions presented in the study are included in the article/Supplementary Materials; further inquiries can be directed to the corresponding authors.

## Supplementary Materials

The supporting information can be downloaded at: https://www.mdpi.com/article/10.3390/ijms252313026/s1.

## References

[1] Karamanos N.K., Theocharis A.D., Piperigkou Z., Manou D., Passi A., Skandalis S.S., Vynios D.H., Orian-Rousseau V., Ricard-Blum S., Schmelzer C.E.H. (2021). A Guide to the Composition and Functions of the Extracellular Matrix. FEBS J..

[2] Lloyd S.M., He Y. (2024). Exploring Extracellular Matrix Crosslinking as a Therapeutic Approach to Fibrosis. Cells.

[3] Ma H.-Y., Li Q., Wong W.R., N’Diaye E.-N., Caplazi P., Bender H., Huang Z., Arlantico A., Jeet S., Wong A. (2023). LOXL4, but Not LOXL2, Is the Critical Determinant of Pathological Collagen Cross-Linking and Fibrosis in the Lung. Sci. Adv..

[4] Jones M.G., Andriotis O.G., Roberts J.J., Lunn K., Tear V.J., Cao L., Ask K., Smart D.E., Bonfanti A., Johnson P. (2018). Nanoscale Dysregulation of Collagen Structure-Function Disrupts Mechano-Homeostasis and Mediates Pulmonary Fibrosis. eLife.

[5] Lyu C., Kong W., Liu Z., Wang S., Zhao P., Liang K., Niu Y., Yang W., Xiang C., Hu X. (2023). Advanced Glycation End-Products as Mediators of the Aberrant Crosslinking of Extracellular Matrix in Scarred Liver Tissue. Nat. Biomed. Eng..

[6] Maller O., Drain A.P., Barrett A.S., Borgquist S., Ruffell B., Zakharevich I., Pham T.T., Gruosso T., Kuasne H., Lakins J.N. (2021). Tumour-Associated Macrophages Drive Stromal Cell-Dependent Collagen Crosslinking and Stiffening to Promote Breast Cancer Aggression. Nat. Mater..

[7] Guo Q., Sun D., Barrett A.S., Jindal S., Pennock N.D., Conklin M.W., Xia Z., Mitchell E., Samatham R., Mirza N. (2022). Mammary Collagen Is under Reproductive Control with Implications for Breast Cancer. Matrix Biol..

[8] Yoshida K., Jiang H., Kim M., Vink J., Cremers S., Paik D., Wapner R., Mahendroo M., Myers K. (2014). Quantitative Evaluation of Collagen Crosslinks and Corresponding Tensile Mechanical Properties in Mouse Cervical Tissue during Normal Pregnancy. PLoS ONE.

[9] Stammers M., Ivanova I.M., Niewczas I.S., Segonds-Pichon A., Streeter M., Spiegel D.A., Clark J. (2020). Age-Related Changes in the Physical Properties, Cross-Linking, and Glycation of Collagen from Mouse Tail Tendon. J. Biol. Chem..

[10] Naffa R., Holmes G., Ahn M., Harding D., Norris G. (2016). Liquid Chromatography-Electrospray Ionization Mass Spectrometry for the Simultaneous Quantitation of Collagen and Elastin Crosslinks. J. Chromatogr. A.

[11] Takaoka A., Babar N., Hogan J., Kim M., Price M.O., Price F.W., Trokel S.L., Paik D.C. (2016). An Evaluation of Lysyl Oxidase–Derived Cross-Linking in Keratoconus by Liquid Chromatography/Mass Spectrometry. Investig. Ophthalmol. Vis. Sci..

[12] Yamauchi M., Taga Y., Hattori S., Shiiba M., Terajima M. (2018). Chapter 6 Analysis of Collagen and Elastin Cross-Links. Methods Cell Biol..

[13] Yao Y., Findlay A., Stolp J., Rayner B., Ask K., Jarolimek W. (2022). Pan-Lysyl Oxidase Inhibitor PXS-5505 Ameliorates Multiple-Organ Fibrosis by Inhibiting Collagen Crosslinks in Rodent Models of Systemic Sclerosis. Int. J. Mol. Sci..

[14] Molnar A., Lakat T., Hosszu A., Szebeni B., Balogh A., Orfi L., Szabo A.J., Fekete A., Hodrea J. (2021). Lyophilization and Homogenization of Biological Samples Improves Reproducibility and Reduces Standard Deviation in Molecular Biology Techniques. Amino Acids.

[15] Chaudhari N., Findlay A.D., Stevenson A.W., Clemons T.D., Yao Y., Joshi A., Sayyar S., Wallace G., Rea S., Toshniwal P. (2022). Topical Application of an Irreversible Small Molecule Inhibitor of Lysyl Oxidases Ameliorates Skin Scarring and Fibrosis. Nat. Commun..

[16] Tang J.C.Y., Dutton J.J., Piec I., Green D., Fisher E., Washbourne C.J., Fraser W.D. (2016). LC–MS/MS Application for Urine Free Pyridinoline and Free Deoxypyridinoline: Urine Markers of Collagen and Bone Degradation. Clin. Mass Spectrom..

[17] Williams M.L., Olomukoro A.A., Emmons R.V., Godage N.H., Gionfriddo E. (2023). Matrix Effects Demystified: Strategies for Resolving Challenges in Analytical Separations of Complex Samples. J. Sep. Sci..

[18] Houghton R., Pita C.H., Ward I., Macarthur R. (2009). Generic Approach to Validation of Small-Molecule LCMS/MS Biomarker Assays. Bioanalysis.

[19] Gao S., Bhoopathy S., Zhang Z.-P., Wright D.S., Jenkins R., Karnes H.T. (2006). Evaluation of Volatile Ion-Pair Reagents for the Liquid Chromatography–Mass Spectrometry Analysis of Polar Compounds and Its Application to the Determination of Methadone in Human Plasma. J. Pharm. Biomed. Anal..

[20] Stoilov I., Starcher B.C., Mecham R.P., Broekelmann T.J. (2018). Chapter 7 Measurement of Elastin, Collagen, and Total Protein Levels in Tissues. Methods Cell Biol..

[21] Fu K., Corbley M.J., Sun L., Friedman J.E., Shan F., Papadatos J.L., Costa D., Lutterodt F., Sweigard H., Bowes S. (2008). SM16, an Orally Active TGF-&bgr; Type I Receptor Inhibitor Prevents Myofibroblast Induction and Vascular Fibrosis in the Rat Carotid Injury Model. Arter. Thromb. Vasc. Biol..

[22] Fuentes-Lemus E., Hägglund P., López-Alarcón C., Davies M.J. (2021). Oxidative Crosslinking of Peptides and Proteins: Mechanisms of Formation, Detection, Characterization and Quantification. Molecules.

