# Supplementary material for: Rapid LC-MS/MS Evaluation of Collagen and Elastin Crosslinks in Human and Mouse Lung Tissue with a Novel Bioanalytical Surrogate Matrix Approach
Source: Int J Mol Sci. 2024 Dec 4;25(23):13026. doi: 10.3390/ijms252313026 (PMC11641751; doi:10.3390/ijms252313026)
Supplement: Supplementary file 1 [file ijms-25-13026-s001.zip › ijms-3288075-supplementary.pdf]

Rapid LC-MS/MS Evaluation of Collagen and Elastin Crosslinks in Human and Mouse Lung Tissue with a Novel Bioanalytical Surrogate Matrix Approach

Sarah M. Lloyd, Elizabeth J. Sande, Kenneth Ruterbories, Stephen P. O'Brien, Yue-Ting Wang, Lucy A. Phillips, Tracy L. Carr, Meghan Clements, Lisa A. Hazelwood, Yu Tian, Yupeng He and Qin C. Ji

Supplementary Material

A. **B.**

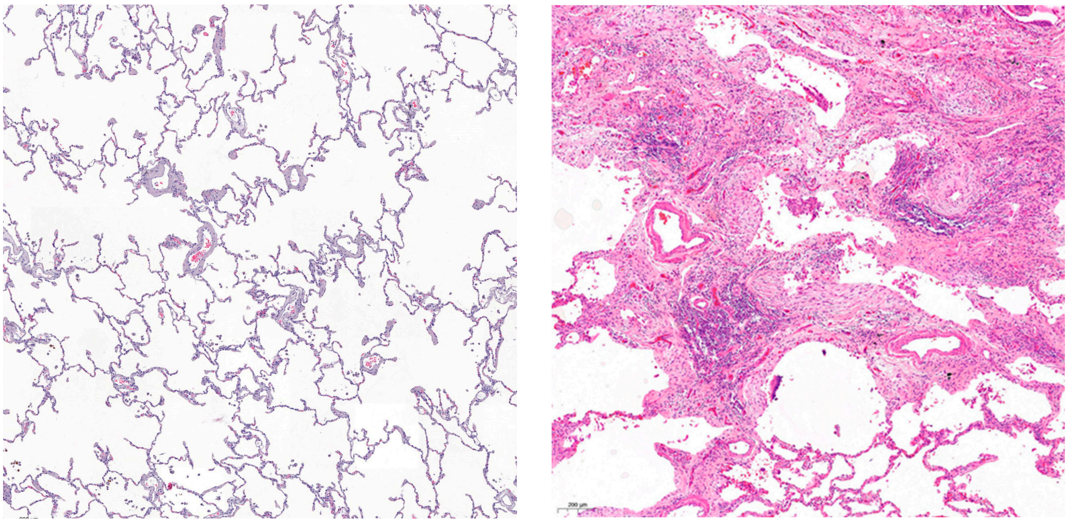

Figure S1. Histological assessment of human lung samples. **(A)** Healthy donor lung tissue **(B)** Idiopathic Pulmonary Fibrosis (IPF) lung tissue.

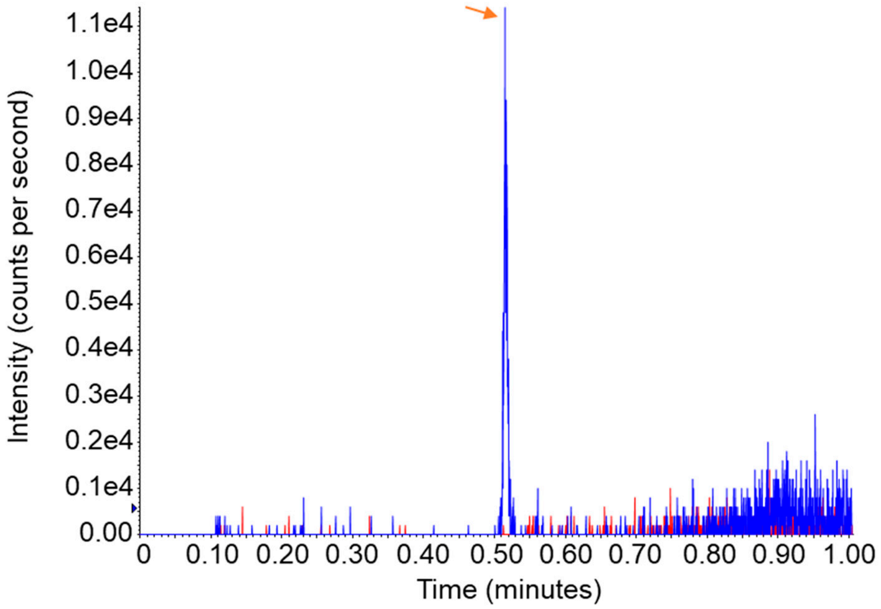

Figure S2. Chromatogram of D4-DHLNL standard with Atmospheric Pressure Chemical Ionization (APCI) strategy yields low intensity signal. Orange arrow points to expected peak location for D4-DHLNL.
